# Phenotypically Selective Genotyping Realizes More Genetic Gains in a Rainbow Trout Breeding Program in the Presence of Genotype-by-Environment Interactions

**DOI:** 10.3389/fgene.2020.00866

**Published:** 2020-09-11

**Authors:** Thinh Tuan Chu, Anders Christian Sørensen, Mogens Sandø Lund, Kristian Meier, Torben Nielsen, Guosheng Su

**Affiliations:** ^1^Center for Quantitative Genetics and Genomics, Aarhus University, Tjele, Denmark; ^2^Department of Animal Breeding and Genetics, Faculty of Animal Science, Vietnam National University of Agriculture, Hanoi, Vietnam; ^3^Danmarks Center for Vildlaks, Randers, Denmark; ^4^AquaSearch ova, Billund, Denmark

**Keywords:** selective genotyping, genomic selection, breeding program design, genotype-by-environment interactions, rainbow trout, fish

## Abstract

Selective genotyping of phenotypically superior animals may lead to bias and less accurate genomic breeding values (GEBV). Performing selective genotyping based on phenotypes measured in the breeding environment (B) is not necessarily a good strategy when the aim of a breeding program is to improve animals’ performance in the commercial environment (C). Our simulation study compared different genotyping strategies for selection candidates and for fish in C in a breeding program for rainbow trout in the presence of genotype-by-environment interactions when the program had limited genotyping resources and unregistered pedigrees of individuals. For the reference population, selective genotyping of top and bottom individuals in C based on phenotypes measured in C led to the highest genetic gains, followed by random genotyping and then selective genotyping of top individuals in C. For selection candidates, selective genotyping of top individuals in B based on phenotypes measured in B led to the highest genetic gains, followed by selective genotyping of top and bottom individuals and then random genotyping. Selective genotyping led to bias in predicting GEBV. However, in scenarios that used selective genotyping of top fish in B and random genotyping of fish in C, predictions of GEBV were unbiased, with genetic correlations of 0.2 and 0.5 between traits measured in B and C. Estimates of variance components were sensitive to genotyping strategy, with an overestimation of the variance with selective genotyping of top and bottom fish and an underestimation of the variance with selective genotyping of top fish. Unbiased estimates of variance components were obtained when fish in B and C were genotyped at random. In conclusion, we recommend phenotypic genotyping of top and bottom fish in C and top fish in B for the purpose of selecting breeding animals and random genotyping of individuals in B and C for the purpose of estimating variance components when a genomic breeding program for rainbow trout aims to improve animals’ performance in C.

## Introduction

In many breeding programs, genotyping is limited to phenotypically superior animals, referred to as selective genotyping of top animals. Such selective genotyping leads to biased predictions of genomic breeding values (GEBV) when genomic-based best linear unbiased prediction (GBLUP) is used (Gowane et al., 2019; Wang et al., 2020). For example, Wang et al. (2020) showed that the use of a combined matrix (Christensen and Lund, 2010; Aguilar et al., 2011) of pedigree and genomic relationships in a single-step GBLUP (ssGBLUP) prediction resulted in the upward bias of GEBV and overestimation of variance components when only a proportion of top individuals were genotyped. The bias in variance estimates and GEBV increased as the proportion of top individuals genotyped increased. Gowane et al. (2019) also showed that with selective genotyping of top animals, the use of a genomic relationship matrix in a GBLUP prediction led to biased GEBV, but the use of a combined relationship matrix (Christensen and Lund, 2010; Aguilar et al., 2011) constructed from pedigree and genomic information resulted in the unbiased prediction of GEBV. Compared to random genotyping or selective genotyping of phenotypically contrasting animals, selective genotyping of top animals in a reference population for training genomic selection models less accurately predicted GEBV (Boligon et al., 2012; Gowane et al., 2019). According to Gowane et al. (2019), genotyping of phenotypically contrasting animals (selective genotyping of top and bottom animals) for selection candidates is superior to selective genotyping of top animals. The undesirable consequences of selectively genotyping top animals have been addressed extensively (VanRaden et al., 2009; Patry and Ducrocq, 2011; Vitezica et al., 2011; Boligon et al., 2012; Jiménez-Montero et al., 2012; Chu et al., 2019; Gowane et al., 2019; Wang et al., 2020), but the superiority of this selective strategy for breeding programs has been shown only by Howard et al. (2018). In addition, some simulation studies (Patry and Ducrocq, 2011; Vitezica et al., 2011; Boligon et al., 2012; Gowane et al., 2019) have used the correlation between true breeding values (TBV) and GEBV to compare different genotyping strategies. However, the difference in correlations between different genotyping strategies may not be consistent with the difference in realized genetic gains, because other factors – such as the intensity of selection, prediction bias, and changes in variance due to selection – may affect these gains. These studies (Patry and Ducrocq, 2011; Vitezica et al., 2011; Boligon et al., 2012; Gowane et al., 2019) did not account for selection, and the effects of bias on genetic gains in breeding programs were not investigated for different genotyping strategies. Therefore, in a comparison of genotyping strategies, gains in genetic merits in a breeding program may be a better measure of assessment.

Comparisons of genotyping strategies have focused on species other than rainbow trout (Boligon et al., 2012; Howard et al., 2018; Gowane et al., 2019; Wang et al., 2020). In two studies (Boligon et al., 2012; Gowane et al., 2019), different genotyping strategies based on phenotypes were used with individuals in the reference population, but the animals in the validation population did not have phenotypes, and thus, random genotyping was used with these validation individuals. However, as the phenotypes of validation individuals or selection candidates can be obtained before genotyping, selective genotyping can also be used with these fish in a breeding program for trout. Other features of trout breeding programs include high fecundity, the use of factorial mating, the ability to control the sex ratios of offspring with sex reversal technology, and the high cost of registering the pedigrees of individuals in the whole population. The high fecundity of trout can translate into a high intensity of selection and highly selective genotyping of reference and validation populations. Because of the high cost, the pedigrees of non-genotyped individuals may not be registered, and phenotypes of these individuals are thus not used to predict EBV. It is unknown whether these features of breeding programs for trout exacerbate prediction bias or lower the accuracy of selection when selective genotyping of top animals occurs. The effects of different selective genotyping strategies on variance components have not been shown when pedigree information is missing.

Environmental differences between nucleus breeding stocks (B) and commercial production farms (C) may lead to genotype-by-environment (G×E) interactions (i.e., the best genotypes in B may not be the best in C). Strong G×E interactions due to environmental differences were found in a breeding program for trout, with the correlations of 0.09–0.58 between traits measured in B and C (Kause et al., 2005). Under this strong re-ranking situation, a sib-testing scheme is required for breeding to improve the performance of animals in C (Mulder and Bijma, 2005; Chu et al., 2018). In a sib-testing scheme, selection candidates are kept for phenotype testing in B, whereas their sibs are transferred to C for phenotype testing (Chu et al., 2019). The individuals in C are then used as a reference population to predict the GEBV of the candidates. Studies (Boligon et al., 2012; Gowane et al., 2019) have shown that in the reference population, predictive accuracy was best with genotyping of phenotypically contrasting animals, followed by random genotyping and then selective genotyping of top animals. However, it is not known which genotyping strategy is best for selection candidates when the breeding program aims to improve animals’ performance in C and selection for genotyping of candidates is based on phenotype measured in B.

We compared genomic selection breeding schemes for trout when G×E interactions were present, genotyping effort was limited and pedigree was not registered. We investigated (1) genotyping strategies of selection candidates based on phenotype measured in B; (2) genotyping strategies of fish in the reference population based on phenotype measured in C; (3) proportions of genotyping allocated to fish in B *versus* C; (4) overlapping genetic makeup among years of selection; (5) the magnitude of G×E interactions; and (6) heritability of the trait.

## Materials and Methods

### Design of the Simulation

The stochastic simulation program ADAM (Pedersen et al., 2009) was used to simulate sib-testing breeding schemes for trout in B and C. The founder population for the simulated breeding schemes consisted of 958 genotyped rainbow trout from AquaSearch ova Aps, Billund, Denmark. After quality control, imputation, and phasing by AquaGen AS, Trondheim, Norway, the genotypes of the fish had 36,451 SNP markers. From these SNPs, 3742 randomly chosen loci were assigned as QTL for the simulation of traits, and the remaining 32,709 loci were used as markers for genomic prediction. The genome, with a total length of 2927.1 cM, consisted of 29 pairs of chromosomes. The trait measured in B and C was taken as two correlated traits controlled fully by the QTL.

Simulation of the trait and individual phenotypes was detailed previously (Chu et al., 2018). The mean (=0), genetic variance (=1), and heritability (*h*^2^) of the trait in the founder population were assumed to be identical for the trait measured in B as for the trait measured in C. Breeding schemes were run for seven overlapping generations that were equivalent to 21 years (*t* = 1, …, 21). In year *t* = 1, …, 3, we randomly selected sires and dams from the base population that was created by sampling haplotypes from the founder generation. It took 3 years for offspring to be phenotyped, genotyped, and sexually mature. In years *t* = 4, …, 21, the selection of males and females was based on GBLUP. In some scenarios when females were not genotyped, phenotypic selection of the females was used. In breeding for rainbow trout, mating time can be manipulated precisely in sexually mature males, whereas the spawning time of females cannot be fully controlled. In addition, males, known as neo-males or sex-reversed males, can produce sperm only once, whereas females can spawn over several years.

In the simulation, 50 males (3 years old) were selected as sires for mating each year, and 400 females (3 or 4 years old) were selected as potential dams for mating. The 3-year-old females were selected from among selection candidates that were 3 years old. The 4-year-old females were selected from among the 3-year-old females selected in the previous year. The proportion of selected females that were kept in 2 consecutive years was a factor we investigated and thus varied by scenario. In total, 400 selected females from which only 50 dams were randomly chosen for mating needed to be available each year. Each year, 50 sires and 50 dams were used for partly factorial mating: sires were mated to two different dams each, and dams were mated to two different sires each. This partly factorial mating, described in Su et al. (2020), resulted in the creation of 100 full-sib families of family size 200 giving 20,000 offspring per year. The number of offspring distributed to B and C varied by scenario. Fish could have phenotypic records measured in either B or C, but not all 20,000 fish were phenotyped and genotyped. Each year, 1000 individuals with phenotypes, including both B and C fish, were genotyped.

### Factors Investigated

The six factors investigated in this study were genotyping of fish in C, genotyping of fish in B, proportions of genotyping allocated to fish in B *versus* C, the proportion of selected females kept in 2 consecutive years, the genetic correlation (*r*_*g*_) between trait records obtained in B and C, and the heritability of the trait (*h*^2^); see Table 1.

**TABLE 1:** Factors investigated in the simulated breeding program.

**Factor**	**Levels**
Genotyping of fish in a commercial environment (C)	[Random genotyping]; selective genotyping of top fish; selective genotyping of top and bottom fish at two intensities (T1_1B1_1 and T1_2B1_2)
Genotyping of fish in a breeding environment (B)	Random genotyping; [selective genotyping of top fish]; selective genotyping of top and bottom fish at using different numbers of top fish (T1_2B1_2 and T3_4B1_4)
Genetic correlation *r*_*g*_ between trait records obtained in B and C (*r*_*g*_)	[0.2; 0.5; 0.8]
Heritability *h*^2^ of the trait (*h*^2^)	[0.1; 0.3]
Proportion of genotyping allocated to fish in C (%)	0; [20]; 40; 60
Proportion of selected females kept in 2 consecutive years (%)	11; [100]

*Square brackets indicate the levels used in the base scenarios.*

Genotyping of fish in C included the following:

1. Random genotyping: Fish were selected for genotyping at random.
2. Selective genotyping of top fish: The individual with the best phenotype from each random sample of 20 fish was selected for genotyping. The phenotype consisted of trait records measured in C.
3. Selective genotyping of top and bottom fish: Two strategies (T1_1B1_1 and T1_2B1_2) were used. For T1_1B1_1, the individual with the best phenotype and the individual with the worst phenotype from each random sample of 20 fish were selected for genotyping. For T1_2B1_2, top and bottom fish were selected from each random sample of 20 fish in the same way as T1_1B1_1. However, not all the selected top and bottom fish were genotyped because with T1_2B1_2, we selected one individual with the best phenotype from each random sample of two top fish, and one individual with the worst phenotype from each random sample of two bottom fish for genotyping.

Genotyping of fish in B included the following:

1. Random genotyping and selective genotyping of top fish similar to genotyping of fish in C, except that selection was based on trait records measured in B.
2. Selective genotyping of top and bottom fish: Two strategies (T1_2B1_2 and T3_4B1_4) were used. The T1_2B1_2 strategy was the same as described for fish in C. For T3_4B1_4, top and bottom fish were selected from each random sample of 20 fish in the same way as T1_1B1_1. However, not all the selected top and bottom fish were genotyped because with T3_4B1_4, we selected three individuals with the best phenotype from each random sample of four top fish, and one individual with the worst phenotype from each random sample of four bottom fish for genotyping.

Pedigrees and phenotypes of non-genotyped individuals were not registered. For random genotyping in B and C, fish to be genotyped were randomly sampled from all fish available in B and C, respectively. Due to the practicality of fish breeding, the selection for genotyping was based on random sets of 20 fish instead of ranking all individuals to select from. The 20 fish in each random sample were not resampled; thus, each fish had only one chance of being selected for genotyping. The proportions of genotyping allocated to fish in B *versus* C were equivalent to the proportions of offspring distributed to B *versus* C each year. *r*_*g*_ represents magnitudes of G×E interactions, from weak to strong. *h*^2^ was assumed to be the same for B and C. *r*_*g*_ and *h*^2^ were used to simulate the trait in the founder population, as described previously (Chu et al., 2018).

The different proportions of selected females kept in 2 consecutive years were used to investigate the effects of different overlapping genetic makeup among years of selection and the intensity of selection. Each year 400 females were needed to be available for mating. When 11% of selected females were used in 2 consecutive years, 360 females were selected from among the 3-year-old offspring in the current year, and 40 individuals (4 years old) were selected from among the 360 selected females in the previous year. On average, 10 and 90% of the maternal genetic makeup of offspring in a year was from 4-year-old dams and 3-year-old dams, respectively. When 100% of selected females were used in 2 consecutive years, 200 females were selected from among the 3-year-old offspring in the current year, and all 200 selected females were kept the following year. In this case, an average of 50 and 50% of the maternal genetic makeup of offspring in a year was from 4-year-old dams and 3-year-old dams, respectively.

Not all combinations of factors were investigated, but base scenarios and their alternatives were. For example, to investigate different genotyping strategies applied to fish in C, schemes considered selective genotyping of top fish in B; *r*_*g*_ of 0.2, 0.5, and 0.8; *h*^2^ of 0.1 and 0.3; 20% of genotyping allocated to fish in C; and 100% of selected females kept in 2 consecutive years. However, in the scenarios that used different proportions of genotyping allocated to fish in C and different proportions of selected females kept in 2 consecutive years, only *h*^2^ of 0.3 was considered.

### Statistical Model

The breeding goal had an economic value of 1 for the performance of fish in C and an economic value of 0 for the performance of fish in B. For scenarios that had genotyped fish in C, we predicted GEBV using the following bivariate GBLUP model:

(1) $$\left[\begin{array}{c} {\text{y}}_{B}\\ {\text{y}}_{C} \end{array}\right]=\left[\begin{array}{cc} {\text{X}}_{B} & 0\\ 0 & {\text{X}}_{C} \end{array}\right]\,\left[\begin{array}{c} {\text{b}}_{B}\\ {\text{b}}_{C} \end{array}\right]+\left[\begin{array}{cc} {\text{Z}}_{B} & 0\\ 0 & {\text{Z}}_{C} \end{array}\right]\,\left[\begin{array}{c} {\text{g}}_{B}\\ {\text{g}}_{C} \end{array}\right]+\left[\begin{array}{c} {\text{e}}_{B}\\ {\text{e}}_{C} \end{array}\right]$$

where **y_B_** and **y_C_** are vectors of phenotypic records of fish in B and C; **b_B_** and **b_C_** are vectors of the fixed effects of year for records in B and C; **g_B_** and **g_C_** are vectors of breeding values of B and C performance, which were assumed to follow the multivariate normal distribution

$$\left[\begin{array}{c} {\text{g}}_{B}\\ {\text{g}}_{C} \end{array}\right]∼M\,V\,N\,\left[0,\text{G}⊗\left(\begin{array}{cc} \sigma_{g_{B}}^{2} & \sigma_{g\,B\,g\,C}\\ \sigma_{g\,C\,g\,B} & \sigma_{g_{C}}^{2} \end{array}\right)\right]$$

where **G** is a genomic relationship matrix constructed based on marker data, ⊗ is the Kronecker product, $\sigma_{g_{B}}^{2}$ and $\sigma_{g_{C}}^{2}$ are the additive genetic variance of trait performance in B and C, respectively, and **σ**_*gBgC*_ is the additive genetic covariance between the two trait performances; **X_B_**, and **Z_B_**, and **X_C_**, and **Z_C_** are incidence matrices associating fixed effects and breeding values with phenotypic records in B and C; and **e_B_** and **e_C_** are vectors of random residuals in B and C, respectively. Model (1) assumed

$$\left[\begin{array}{c} {\text{e}}_{B}\\ {\text{e}}_{C} \end{array}\right]∼M\,V\,N\,\left[0,\left(\begin{array}{cc} {\text{I}}_{B}\,\sigma_{e_{B}}^{2} & 0\\ 0 & {\text{I}}_{C}\,\sigma_{e_{C}}^{2} \end{array}\right)\right]$$

where **I_B_** and **I_C_** are identity matrices corresponding to fish in B and C, respectively, and $\sigma_{e_{B}}^{2}$ and $\sigma_{e_{C}}^{2}$ are the environmental variance of B and C traits, respectively.

For scenarios without records measured in C, we estimated GEBV using a univariate GBLUP model:

(2) $${\text{y}}_{B}={\text{X}}_{B}\,{\text{b}}_{B}+{\text{Z}}_{B}\,{\text{g}}_{B}+{\text{e}}_{B}$$

The description of notations for model (2) is similar to that of model (1), except model (2) is a single-trait model. The selection of scenarios without records measured in C was based on the GEBV of the B trait only.

Each year, GEBV were predicted for all genotyped individuals after all records of genotyped individuals in that year were obtained. Models (1) and (2) used the true genetic variance components to predict GEBV. Computations were performed with the DMU4 module of the DMU package (Madsen and Jensen, 2013).

### Selection and Intensity of Selection

Each year, 1000 fish in B and C were genotyped; 50 sires and up to 360 selected females were needed to restock for breeding. When the proportion of genotyping allocated to fish in C increased, the intensity of selection of fish in B based on GEBV decreased. For example, when 60% of genotyping was allocated to fish in C, only 400 selection candidates in B were genotyped. Genotyping of different sex ratios in C did not affect the intensity of selection or predictive accuracy. However, sex ratio genotyping of candidates in B could have had significant effects on the intensity of selection and thus the genetic gains of a breeding scheme. For a fair comparison of breeding schemes, we used the sex ratio genotyping that would lead to the optimal selection intensity for the scheme. The approach to identifying the optimal selection intensity of a scheme is shown in Appendix 1. We assumed that the sex ratios of the offspring and the genotyped fish in B could be easily manipulated with sex reversal technology and that the trout would have a high reproductive capacity. The sex ratios genotyped for all scenarios can be found in Appendix 2. In the scenarios in which females were not genotyped, the selection of females was based on phenotype in B. The procedures for selecting these females were similar to the selective genotyping of top fish, in which the individual with the best phenotype from each random sample of 20 fish was selected. The selection of breeding females to be kept the following year was random. When the selected females were genotyped, the selection of breeding females kept the following year was based on GEBV.

### Simulation Outputs

For each scenario, 100 replicates were simulated. Means and standard errors of the 100 replicates of were calculated to assess the rate of genetic gain, rate of inbreeding, accuracy of GEBV, and prediction bias. Differences between genetic levels at years 5–7 and 19–21 were used to calculate the rate of genetic gain per year (Δ*G*): Δ*G*=$\frac{\left(G_{19}+G_{20}+G_{21}\right)-\left(G_{5}+G_{6}+G_{7}\right)}{\left(19+20+21\right)-\left(5+6+7\right)}$, where *G*_5_,*G*_6_,*G*_7_,*G*_19_,*G*_20_, and *G*_*21*_ are the average TBV of the C trait of all fish born at years 5, 6, 7, 19, 20, and 21, respectively. The rate of inbreeding per generation (Δ*F*) for a replicate was calculated as Δ*F*  (%) = (1−*e*^β^) ^∗^100, where β is the slope of the linear regression of ln(1−*F*_*t*_) on the generation corresponding to years 5–21 and *F*_*t*_ is the inbreeding coefficient of all fish born at time step *t* based on the pedigree relationship (Hinrichs et al., 2007).

In the scenarios with records measured in C, the accuracy of GEBV was calculated as the correlation between the GEBV of the C trait and the TBV of the C trait for all 3-year-old genotyped B fish at years 10–12. The correlation was calculated for each year from GEBV obtained during that year. The accuracy of GEBV for each replicate was the average of the correlations of years 10–12. The accuracy had the expected value of 1. Similarly, the bias of GEBV was calculated as the regression slope of the TBV of the C trait on the GEBV of the C trait. The bias had the expected value of 1.

In the scenarios without records measured in C, only the GEBV of the B trait were available. The accuracy of GEBV was calculated as the correlation between the GEBV of the B trait and the TBV of the C trait for all 3-year-old genotyped B fish at years 10–12 because the selection to improve performance in C was based solely on the GEBV of the B trait. The accuracy in this situation had the expected value of *r*_*g*_. The bias of GEBV was calculated as the regression slope of the TBV of the C trait on the GEBV of the B trait. The bias had the expected value of $\left(r_{g}\times \sqrt{\frac{\sigma_{g_{C}}^{2}}{\sigma_{g_{B}}^{2}}}\right)$ (see the derivations of the expected values in Appendix 3). As $\sigma_{g_{B}}^{2}$ and $\sigma_{g_{C}}^{2}$ were identical, and equal to 1, the expected bias of GEBV was *r*_*g*_ in the scenarios without records measured in C.

Because of computational challenges, we estimated variance components for select scenarios only at year *t* = 10. The estimation of variance components used the DMUAI module of the DMU package (Madsen and Jensen, 2013). Estimated variance components at year *t* = 10 were not used to predict GEBV in the following year of the breeding scenarios.

## Results

**FIGURE 1:**
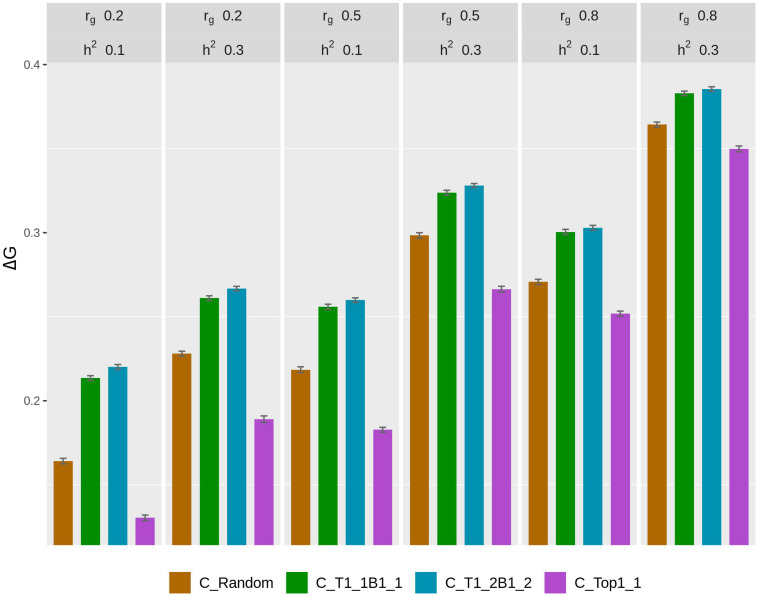
Rate of genetic gain (Δ*G*; mean of 100 replicates ± standard error) of different genotyping scenarios for 200 fish in C: random genotyping (C_Random); selection of the phenotypically best and worst fish from each sample of 20 fish (C_T1_1B1_1); selection of fish from one of the two top fish and one of the two bottom fish, where the top and bottom fish were the best and worst fish, respectively, from each sample of 20 fish (C_T1_2B1_2); and selection of the phenotypically best fish from each sample of 20 fish (C_Top1_1). The scenarios assumed genetic correlations (*r*_*g*_) between the trait measured in B and C of 0.2, 0.5, and 0.8 and heritability of the trait of 0.1 and 0.3. The scenarios used selective genotyping of top fish for selection candidates in B, and 100% of selected females were kept in 2 consecutive years. C, commercial environment; B, breeding environment.

The rate of genetic gain, accuracy of GEBV, prediction bias, and rate of inbreeding for breeding scenarios that used different genotyping strategies for fish in C are presented in Figure 1 and Table 2. The scenarios used selective genotyping of top fish for selection candidates in B, and 100% of selected females were kept in 2 consecutive years. The scenarios with selective genotyping of top and bottom fish in C led to the highest Δ*G*, followed by random genotyping and then selective genotyping of top fish. Genetic gains increased when the phenotypic differentiation of genotyped fish in C increased. Genetic gains were highest with T1_2B1_2. The difference in accuracy of GEBV between the scenarios followed a similar trend as Δ*G*. Selective genotyping of top fish led to not only the least accurate GEBV but deflated predictions of GEBV. Selective genotyping of top and bottom fish in C led to inflated predictions of GEBV. Prediction bias was greater when selection for genotyping was more intense (i.e., in T1_2B1_2 compared to T1_1B1_1).

**TABLE 2:** Accuracy of GEBV, prediction bias, and rate of inbreeding (mean of 100 replicates) for different genotyping scenarios for fish in C.

	***h*^2^ = 0.1**				***h*^2^ = 0.3**			
***r*_*g*_**	**Random**	**T1_1B1_1**	**T1_2B1_2**	**Top1_1**	**Random**	**T1_1B1_1**	**T1_2B1_2**	**Top1_1**
**Accuracy of GEBV**								
0.2	0.362	0.515	0.534	0.245	0.512	0.635	0.656	0.371
0.5	0.389	0.522	0.544	0.284	0.525	0.641	0.661	0.395
0.8	0.436	0.548	0.564	0.363	0.555	0.65	0.666	0.474
SE	0.005	0.004	0.004	0.005	0.004	0.003	0.002	0.005
**Prediction bias**								
0.2	0.961	0.59	0.506	1.232	0.976	0.528	0.465	1.423
0.5	1.045	0.624	0.544	1.324	1.023	0.554	0.484	1.428
0.8	1.207	0.763	0.656	1.434	1.142	0.645	0.573	1.527
SE	0.013	0.005	0.005	0.024	0.008	0.003	0.003	0.017
**Rate of inbreeding**								
0.2	0.729	0.872	0.851	0.643	0.858	0.881	0.901	0.762
0.5	0.842	0.838	0.887	0.646	0.944	0.913	0.963	0.81
0.8	0.761	0.93	0.887	0.674	0.931	0.952	0.975	0.734
SE	0.022	0.030	0.028	0.017	0.031	0.028	0.037	0.027

*Genotyping of fish in C: random (Random); selection of the phenotypically best and worst fish from each sample of 20 fish (T1_1B1_1); selection of fish from one of the two top fish and one of the two bottom fish, where the top and bottom fish were the best and worst fish, respectively, from each sample of 20 fish (T1_2B1_2); and selection of the phenotypically best fish from each sample of 20 fish (Top1_1). *r*_*g*_ is the genetic correlation between the trait measured in B and C. *h*^2^ is the heritability of the trait simulated. The scenarios used selective genotyping of top fish for selection candidates in B, and 100% of selected females were kept in 2 consecutive years. Standard errors (SE) are the average values for different *r*_*g*_. GEBV, genomic breeding values; C, commercial environment; B, breeding environment.*

In terms of Δ*F*, the scenarios with selective genotyping of top and bottom fish in C were less favorable than those with random genotyping and genotyping of top fish. However, when the rate of genetic gain per 1% increase in inbreeding (Δ*G*/Δ*F*) was used as the comparison criterion, the scenarios with selective genotyping of top and bottom fish in C were most favorable. In terms of Δ*G*/Δ*F*, T1_1B1_1 was generally the best genotyping strategy for fish in C.

As *r*_*g*_ increased from 0.2 to 0.8, Δ*G* and the accuracy of GEBV increased for all scenarios for fish in C. Increasing *r*_*g*_ from 0.2 to 0.5 also increased Δ*F* in all scenarios. When *r*_*g*_ increased from 0.5 to 0.8, Δ*F* increased in some scenarios but not others. When *h*^2^ increased from 0.1 to 0.3, Δ*G*, the accuracy of GEBV, and Δ*F* increased. With increasing *r*_*g*_, the prediction bias of GEBV decreased in scenarios that used selective genotyping of top and bottom fish in C. In contrast, with increasing *r*_*g*_, bias increased in scenarios that used random genotyping or selective genotyping of top fish. The difference in Δ*G* between different scenarios for fish in C tended to decrease as *r*_*g*_ increased or *h*^2^ increased.

**FIGURE 2:**
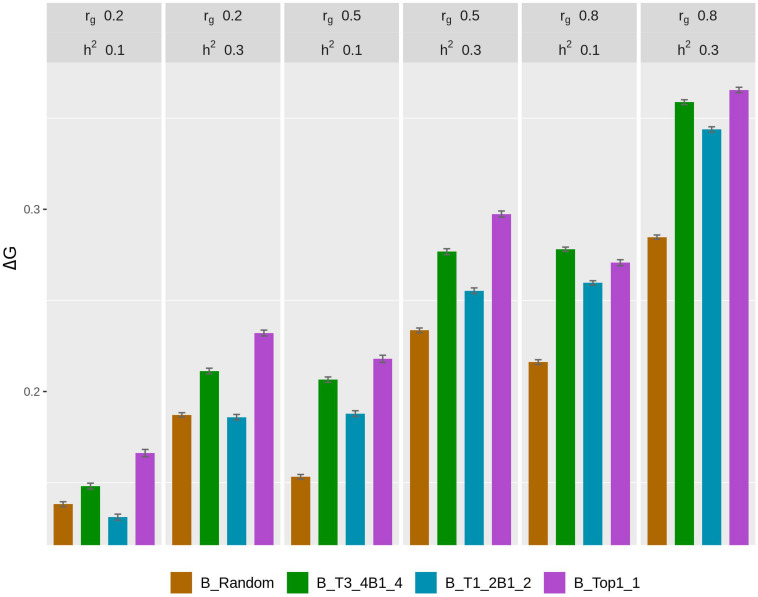
Rate of genetic gain (Δ*G*; mean of 100 replicates ± standard error) of different genotyping scenarios for 800 fish in B: random genotyping (B_Random); selection of fish from three of the four top fish and one of the four bottom fish, where the top and bottom fish were the best and worst fish, respectively, from each sample of 20 fish (B_T3_4B1_4); selection of fish from one of the two top fish and one of the two bottom fish, where the top and bottom fish were the best and worst fish, respectively, from each sample of 20 fish (B_T1_2B1_2); and selection of the phenotypically best fish from each sample of 20 fish (B_Top1_1). The scenarios assumed genetic correlations (*r*_*g*_) between the trait measured in B and C of 0.2, 0.5, and 0.8 and heritability of the trait of 0.1 and 0.3. The scenarios used random genotyping of fish in C, and 100% of selected females were kept in 2 consecutive years. C, commercial environment; B, breeding environment.

The rate of genetic gain, accuracy of GEBV, prediction bias, and rate of inbreeding for breeding scenarios that used different genotyping strategies for fish in B are presented in Figure 2 and Table 3. The scenarios used random genotyping of fish in C, and 100% of selected females were kept in 2 consecutive years. The scenarios with selective genotyping of top fish in B led to the highest Δ*G*, followed by selective genotyping of top and bottom and then random genotyping. Among the selective genotyping strategies, Δ*G* increased when the proportion of top fish genotyped increased. The exception to this was that when *r*_*g*_ = 0.8 and *h*^2^ = 0.1, TB3_4B1_4 led to a higher Δ*G* than selective genotyping of top fish in B. When Δ*G*/Δ*F* and Δ*F* were used as the comparison criteria, selective genotyping of top fish in B was the least favorable genotyping strategy, and random genotyping or TB3_4B1_4 was most favorable. The accuracy of GEBV decreased as the proportion of top fish genotyped increased. T1_2B1_2 for fish in B had the most accurate GEBV, but these scenarios also had the highest prediction bias of GEBV.

**TABLE 3:** Accuracy of GEBV, prediction bias, and rate of inbreeding (mean of 100 replicates) for different genotyping scenarios for fish in B.

	***h*^2^ = 0.1**				***h*^2^ = 0.3**			
***r*_*g*_**	**Random**	**T3_4B1_4**	**T1_2B1_2**	**Top1_1**	**Random**	**T3_4B1_4**	**T1_2B1_2**	**Top1_1**
**Accuracy of GEBV**								
0.2	0.367	0.37	0.391	0.356	0.519	0.527	0.551	0.514
0.5	0.419	0.476	0.502	0.387	0.588	0.626	0.663	0.521
0.8	0.538	0.641	0.68	0.428	0.674	0.777	0.816	0.56
SE	0.004	0.004	0.004	0.005	0.003	0.003	0.003	0.004
**Prediction bias**								
0.2	0.957	0.815	0.764	0.952	0.988	0.902	0.876	0.979
0.5	0.967	0.647	0.552	1.03	0.984	0.746	0.699	1.021
0.8	0.973	0.585	0.49	1.177	0.991	0.657	0.605	1.152
SE	0.009	0.007	0.006	0.014	0.006	0.005	0.004	0.008
**Rate of inbreeding**								
0.2	0.519	0.616	0.642	0.761	0.456	0.783	0.719	0.892
0.5	0.47	0.589	0.697	0.85	0.611	0.679	0.689	0.986
0.8	0.565	0.57	0.67	0.815	0.582	0.588	0.719	0.964
SE	0.012	0.014	0.017	0.029	0.014	0.027	0.020	0.052

*Genotyping of fish in B: random (Random); selection of fish from three of the four top fish and one of the four bottom fish, where the top and bottom fish were the best and worst fish, respectively, from each sample of 20 fish (T3_4B1_4); selection of fish from one of the two top fish and one of the two bottom fish, where the top and bottom fish were the best and worst fish, respectively, from each sample of 20 fish (T1_2B1_2); and selection of the phenotypically best fish from each sample of 20 fish (Top1_1). *r*_*g*_ is the genetic correlation between the trait measured in B and C. *h*^2^ is the heritability of the trait simulated. The scenarios used random genotyping of fish in C, and 100% of selected females were kept in 2 consecutive years. Standard errors (SE) are the average values for different *r*_*g*_. GEBV, genomic breeding values; B, breeding environment; C, commercial environment.*

With *r*_*g*_ of 0.2 and 0.5, prediction bias was negligible in the scenarios that used selective genotyping of top fish in B, whereas with *r*_*g*_ of 0.8 prediction deflated slightly. Among the scenarios that used selective genotyping of fish in B, prediction bias increased as *r*_*g*_ increased or *h*^2^ decreased. Overall, Δ*G*, the accuracy of GEBV, and Δ*F* increased as *r*_*g*_ increased from 0.2 to 0.8 or *h*^2^ increased from 0.1 to 0.3.

**FIGURE 3:**
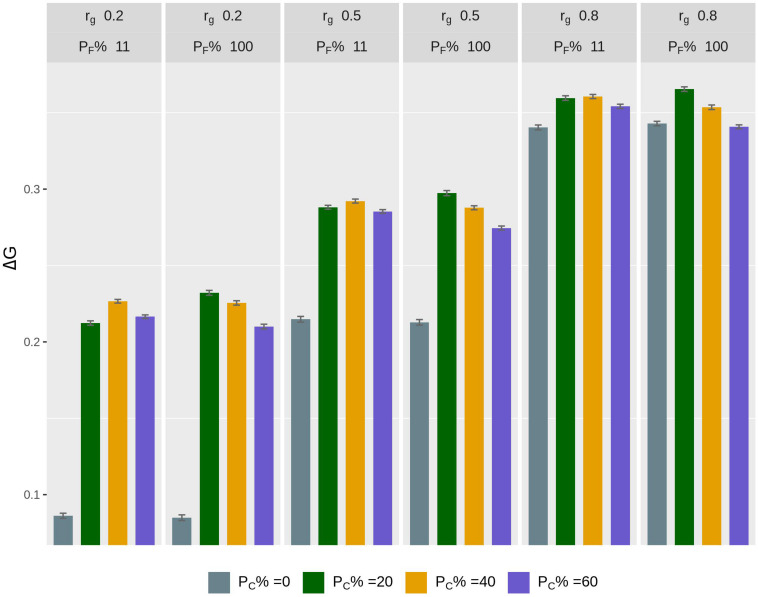
Rate of genetic gain (Δ*G*; mean of 100 replicates ± standard error) of scenarios allocating different proportions of genotyping to fish in C (P_*C*_% = 0, 20, 40, and 60) and keeping different proportions of selected females in 2 consecutive years (P_*F*_% = 100 and 11) for different genetic correlations (*r*_*g*_) between B and C traits. The scenarios assumed heritability of the trait of 0.3 and used selective genotyping of top fish in B and random genotyping of fish in C. C, commercial environment; B, breeding environment.

The rate of genetic gain, accuracy of GEBV, prediction bias, and rate of inbreeding for breeding scenarios that allocated different proportions of genotyping to fish in C and kept different proportions of selected females in 2 consecutive years are presented in Figure 3 and Table 4. When 100% of selected females were kept in 2 consecutive years, allocating 20% of genotyping to fish in C led to the highest Δ*G*. Increasing the proportion of fish in C from 20 to 60% decreased Δ*G*. However, the accuracy of GEBV increased as the proportion of genotyping allocated to fish in C increased from 0% to 60%. Increasing the proportion also decreased prediction bias and increased Δ*G*/Δ*F*. The scenarios without genotyped fish in C had the lowest Δ*G* and the least accurate GEBV compared to the scenarios with fish in C.

**TABLE 4:** Accuracy of GEBV, prediction bias, and rate of inbreeding (mean of 100 replicates) of scenarios allocating different proportions of genotyping to fish in C (P_*C*_% = 0, 20, 40, and 60) and keeping different proportions of selected females in 2 consecutive years (P_*F*_% = 100 and 11) for different levels of genetic correlation (*r*_*g*_) between B and C traits.

	**P_*F*_% = 100**				**P_*F*_% = 11**			
***r*_*g*_**	**P_*C*_% = 0**	**P_*C*_% = 20**	**P_*C*_% = 40**	**P_*C*_% = 60**	**P_*C*_% = 0**	**P_*C*_% = 20**	**P_*C*_% = 40**	**P_*C*_% = 60**
**Accuracy of GEBV**								
0.2	0.095	0.514	0.606	0.679	0.091	0.512	0.625	0.67
0.5	0.235	0.521	0.61	0.675	0.238	0.531	0.624	0.672
0.8	0.416	0.56	0.62	0.676	0.41	0.555	0.632	0.67
SE	0.004	0.004	0.003	0.003	0.004	0.004	0.003	0.002
**Prediction bias**								
0.2	0.324	0.979	0.997	0.988	0.306	0.976	0.99	0.989
0.5	0.768	1.021	1.003	1.007	0.779	1.017	1.013	0.997
0.8	1.252	1.152	1.083	1.049	1.234	1.135	1.072	1.039
SE	0.013	0.008	0.006	0.005	0.013	0.008	0.006	0.005
**Rate of inbreeding**								
0.2	0.821	0.892	0.693	0.64	1.247	1.233	1.343	1.188
0.5	0.865	0.986	0.729	0.678	1.172	1.349	1.477	1.322
0.8	0.879	0.964	0.745	0.757	1.191	1.349	1.553	1.434
SE	0.031	0.052	0.029	0.022	0.032	0.038	0.036	0.029

*The scenarios assumed heritability of the trait of 0.3 with selective genotyping of top fish in B and random genotyping of fish in C. Standard errors (SE) are the average values for different *r*_*g*_. GEBV, genomic breeding values; C, commercial environment; B, breeding environment.*

When 11% of selected females were kept in 2 consecutive years, allocating 40% of genotyping to fish in C led to the highest Δ*G*. As the proportion of genotyping allocated to fish in C increased, the trends for accuracy of GEBV, prediction bias, and Δ*G*/Δ*F* became similar to those for the scenarios that kept 100% of selected females in 2 consecutive years.

The difference in Δ*G*, or accuracy of GEBV, between scenarios that kept different proportions of selected females in 2 consecutive years varied with the proportion of genotyping allocated to fish in C. For example, when 20% of genotyping was allocated to fish in C, the scenario with 100% of selected females kept in 2 consecutive years had a higher Δ*G* than the scenario with 11% of selected females kept. However, when 60% of genotyping was allocated to fish in C, the scenario with 100% of selected females kept had a lower Δ*G*. There was little difference in Δ*G* in the scenarios with 20% of genotyping allocated to fish in C and 100% of selected females kept in 2 consecutive years Δ*G* compared to the scenarios with 40% of genotyping allocated to fish in C and 11% of selected females kept. However, the scenarios that kept 100% of selected females had a lower Δ*F*.

When *r*_*g*_ increased, Δ*G* increased significantly, in particular in the scenarios without fish genotyped in C. The difference in Δ*G* between scenarios with and without fish in C decreased as *r*_*g*_ increased. Increasing *r*_*g*_ increased the regression slope of the TBV of the C trait on the GEBV of the B trait. The regression slope reflects the prediction bias for the scenarios without fish in C.

Table 5 presents variance components estimated from the bivariate GBLUP model for different genotyping of fish in B and C. These scenarios were simulated with *r*_*g*_ of 0.5, *h*^2^ of 0.3, 20% of genotyping allocated to fish in C, and 100% of selected females kept in 2 consecutive years. As expected, the estimates of variance components of the scenarios that used random genotyping of fish in both B and C were close to the simulated values for the trait measured in both B and C. When genotyping of fish in B was random, the additive genetic variance and residual variance were close to the simulated values for the trait measured in B. With selective genotyping of top fish in B, the estimated variance components of the trait measured in B, in particular the additive genetic variance, were substantially lower than the simulated values. As a result, the heritability estimates of the trait measured in B were lower than the simulated values. With selective genotyping of top and bottom fish in B, the variance components of the trait measured in B, in particular the additive genetic variance (and thus the heritability), were overestimated. Similarly, the variance components of the trait measured in C were under- and overestimated, respectively, in the scenarios that used selective genotyping of top fish and selective genotyping of top and bottom fish in C.

**TABLE 5:** Variance components (mean of 100 replicates) estimated from the bivariate model for different genotyping scenarios for fish in B and C.

**Parameter**	**Simulated values**	**B-Random C-Random**	**B-Top1_1 C-Random**	**B-T1_2B1_2 C-Random**	**B-Top1_1 C-T1_1B1_1**	**B-Top1_1 C-Top1_1**
$\sigma_{g_{B}}^{2}$	1	0.966	0.199	7.093	0.194	0.203
$\sigma_{g_{C}}^{2}$	1	0.958	0.955	0.918	6.676	0.187
σ_*gBgC*_	0.5	0.475	0.214	1.247	0.486	0.107
$\sigma_{e_{B}}^{2}$	2.333	2.361	1.067	4.913	1.066	1.066
$\sigma_{e_{C}}^{2}$	2.333	2.4	2.4	2.387	4.195	1.077
$h_{B}^{2}$	0.3	0.29	0.157	0.591	0.153	0.16
$h_{C}^{2}$	0.3	0.285	0.284	0.277	0.614	0.147

*Genotyping: random (Random); selection of fish from one of the two top fish and one of the two bottom fish, where the top and bottom fish were the best and worst fish, respectively, from each sample of 20 fish (T1_2B1_2); selection of the phenotypically best fish from each sample of 20 fish (Top1_1); and selection of the phenotypically best and worst fish from each sample of 20 fish (T1_1B1_1). The direct genetic variance, residual variance, and heritability are $\sigma_{g\,B}^{2}$, $\sigma_{e\,B}^{2}$, and $h_{B}^{2}$, respectively, for the trait measured in B and $\sigma_{g\,C}^{2}$, $\sigma_{e\,C}^{2}$, and $h_{C}^{2}$, respectively, for the trait measured in C. The scenarios were simulated with *r*_*g*_ of 0.5, heritability of 0.3, 20% of genotyping allocated to fish in C, and 100% of selected females kept in 2 consecutive years. B, breeding environment; C, commercial environment.*

## Discussion

In this study, the difference in the accuracy of GEBV between genotyping strategies based on phenotype was similar to other studies (Boligon et al., 2012; Jiménez-Montero et al., 2012; Gowane et al., 2019; Su et al., 2020), that is, selective genotyping of top and bottom fish led to the most accurate GEBV, followed by random genotyping and then selective genotyping of top fish. The difference in accuracy between different genotyping strategies for fish in C was consistent with the difference in genetic gains (i.e., selective genotyping of top and bottom fish in C led to the highest genetic gains).

However, when genotyping strategies based on phenotypes were applied to selection candidates in B, selective genotyping of top fish led to the highest genetic gains. This can be explained by the intensity of genomic selection. The number of genotyped fish in B was the same, and the genotyped individuals with the worst phenotypes measured in B were selection candidates as well. However, it was very unlikely that these bottom fish would be selected as parents. Therefore, selection was less intense in the scenarios with random genotyping and selective genotyping of top and bottom fish than in the scenarios with selective genotyping of top fish. Gowane et al. (2019) concluded that selective genotyping of top and bottom animals for selection candidates is better than selective genotyping of top animals in a breeding program. However, the authors did not take into account the potentially lower intensity of selection when selective genotyping of top and bottom animals is used for candidates.

Compared to random genotyping, selective genotyping generally led to higher rates of inbreeding, but the rates in all scenarios were roughly 1% per generation, which is in line with FAO’s recommendation for animal breeding programs (FAO, 2000). Selective genotyping also led to higher prediction bias of GEBV. Prediction bias in our study refers to the over- or under-dispersion (inflation/deflation) of GEBV with respect to TBV. Legarra and Reverter (2017) stated that prediction bias is more relevant for breeding programs for dairy cattle, in which selection involves a mixture of old and young animals. For example, when prediction bias is less than 1, young animals will have higher but less accurate GEBV than old animals (Legarra and Reverter, 2017). Prediction bias may have little effects on the re-ranking of the GEBV of individuals in breeding programs for pigs, chickens, and fish because the breeding cycle is relatively short, dams and sires are culled quickly, and selection candidates are typically carried out from within the same hatch (Legarra and Reverter, 2017). However, prediction bias can be problematic for these breeding programs when selection is based on multiple traits and bias differs among these traits. In another case of selective genotyping of top animals, when both non-genotyped and genotyped individuals are the selection candidates, it could be unfair to genotyped individuals to compare breeding values. The use of ssGBLUP to estimate variance components and predict EBV leads to severely inflated EBV for non-genotyped individuals in this situation (Wang et al., 2020). In addition, bias may lead to estimates of genetic trends that are higher or lower than the true rate of genetic gain.

Gowane et al. (2019) and Howard et al. (2018) found that the unbiased prediction of GEBV in a breeding program with selective genotyping could be obtained with ssGBLUP. ssGBLUP uses phenotypes of non-genotyped animals and combined pedigree and genomic information to construct a relationship matrix between individuals. However, in our study, the pedigrees and phenotypes of non-genotyped animals were not registered. Therefore, the selection of genotyped individuals was not accounted in the GBLUP model that caused biased predictions of GEBV. When selective genotyping of top fish was used, predictions of GEBV were deflated. When selective genotyping of top and bottom fish was used, predictions were inflated. A similar result for prediction bias was found for selective genotyping in Gowane et al. (2019) and Jiménez-Montero et al. (2012) when only genotyped animals were used in predictions. It is interesting that in our study, predictions were deflated only with *r*_*g*_ of 0.8, not with *r*_*g*_ of 0.2 and 0.5 when selective genotyping of top animals was used for fish in B and random genotyping was used for fish in C. A possible reason for this could be that information on the fish in B made a small contribution to predicting the breeding value of trait performance in C when the genetic correlation between B and C was low.

To obtain unbiased predictions of GEBV with selective genotyping, Gowane et al. (2019) used true variance components in the ssGBLUP model. True variance components were also used to estimate GEBV for selection for all simulated breeding schemes in our study. However, when genotyping is selective, it is difficult to obtain unbiased estimates of the variance components. Wang et al. (2020) showed that the use of ssGBLUP led to an overestimation of variance components when selective genotyping of top animals was used. This stands in contrast to our study, in which variance components were underestimated when the GBLUP model was used in scenarios that involved selective genotyping of top fish. The additive genetic variance was underestimated more severely than the residual variance. Use of the pedigree-based BLUP model without the pedigrees and phenotypes of non-genotyped animals did not improve the estimation of variance components (Appendix 4). To the best of our knowledge, no studies have used the GBLUP model to estimate variance components with selective genotyping of top and bottom animals. Variance components in this situation, in particular the additive genetic variance, were largely overestimated (Table 5). The use of random genotyping of fish in B led to plausible estimates of variance in the trait measured in B, and likewise for C. Random genotyping of fish in both B and C was required to ensure that individuals represented the whole distribution of phenotypes and thus that estimates of variance components of the trait measured in B and C were unbiased when the GBLUP model was used.

As the proportion of genotyping allocated to fish in C increased, the accuracy of the GEBV of the trait measured in C increased. However, because genotyping was limited to 1000 fish per year, increasing the proportion of fish in C decreased the number of fish in B and thus the intensity of selection. The optimal proportion for genetic gains of a breeding scheme can be achieved by balancing between accuracy and intensity. In our study, 20 and 40% with *r*_*g*_ 0.2, 0.5, and 0.8 were close to optimal. In a sib-testing breeding program for broiler chicken, a scheme that placed 30% of animals in C for genotype and phenotype testing with *r*_*g*_ 0.5 and 0.7 was optimal (Chu et al., 2018). With *r*_*g*_ of 0.9, a scheme that placed animals in C for testing showed no increase in genetic gains compared to a scheme that kept all animals in B only (Chu et al., 2018).

We compared two different proportions of selected females kept in 2 consecutive years to investigate the effects of overlapping genetic makeup among years of selection. We expected that the scheme that kept 11% of selected females in 2 consecutive years would have the advantage of capitalizing on genetic progress, whereas the one that kept 100% of selected females in 2 consecutive years would result in a higher intensity of selection and more accurate GEBV. Because of the possibly higher maternal overlapping genetic makeup among years of selection, we expected to find a stronger relationship between fish in the current and previous years in the scheme that kept 100% of selected females, which would thus improve accuracy of selection in the current year. A difference in the intensity of selection was observed between the two schemes. However, there was little difference in terms of the accuracy of GEBV. This might be because only 50 dams from among 400 females were selected to mate in a year. In the scheme that kept 100% of selected females in 2 consecutive years, 3.12 females on average were dams in both years. The relationship coefficient between fish in the current and previous years was relatively weak, at 0.016, for non-inbred, unrelated parents. Such a weak relationship means that there was little advantage, if any, in terms of accuracy of selection for the scheme that kept 100% of selected females compared to the alternative scheme. It should be noted that when 40% of genotyping was allocated to fish in C, the accuracy of GEBV was not comparable between the two schemes, as the selection of females was based on GEBV in one scheme and on phenotypic selection in the other (Appendix 2). Nonetheless, the scheme that kept 100% of selected females in 2 consecutive years was preferred, as it had a lower rate of inbreeding.

## Conclusion

In this study, we compared different genotyping strategies in a breeding program for rainbow trout with limited genotyping efforts and G×E interactions due to differences between B and C. We found that to maximize genetic gains in the breeding program, the best strategy was selective genotyping of top and bottom fish in C and selective genotyping of top fish in B. However, selective genotyping led to biased prediction of GEBV and biased estimates of variance components. Yet selective genotyping of top fish in B and random genotyping of fish in C led to unbiased prediction of GEBV when *r*_*g*_ was 0.2 and 0.5. Random genotyping of fish in B and C was required to obtain plausible, unbiased estimates of variance components. When *r*_*g*_ was 0.2, 0.5, and 0.8, the best scheme allocated 20% of genotyping to fish in C and kept 100% of selected females in 2 consecutive years. We recommend phenotypically selective genotyping of top and bottom fish in C and top fish in B for the purpose of selecting breeding animals, and random genotyping of individuals in B and C for the purpose of estimating variance components when G×E interactions are present in a genomic breeding program for rainbow trout.

## Data Availability Statement

The data that support the findings of this study are available from the corresponding author upon reasonable request. Requests to access the data should be directed to chu.thinh@au.dk.

## Author Contributions

TC, GS, AS, and ML designed and coordinated the study. TN and KM contributed to the design of the breeding schemes. GS and ML contributed to the genotyping and phenotyping methods. TC derived sex ratio genotyping. TC and AS designed and conducted the simulations. TC wrote the manuscript, and all other authors commented on and improved drafts of the manuscript.

## Conflict of Interest

At the time of the study, TN was employed by AquaSearch ova (Denmark).

The remaining authors declare that the research was conducted in the absence of any commercial or financial relationships that could be construed as potential conflicts of interest.

## References

[B1] Aguilar I. Misztal I. Legarra A. Tsuruta S. (2011). Efficient computation of the genomic relationship matrix and other matrices used in single-step evaluation. *J. Anim. Breed. Genet.* 128 422–428. 10.1111/j.1439-0388.2010.00912.x 22059575

[B2] Boligon A. A. Long N. Albuquerque L. G. Weigel K. A. Gianola D. Rosa G. J. M. (2012). Comparison of selective genotyping strategies for prediction of breeding values in a population undergoing selection. *J. Anim. Sci.* 90 4716–4722. 10.2527/jas.2012-4857 23372045

[B3] Christensen O. F. Lund M. S. (2010). Genomic prediction when some animals are not genotyped. *Genet. Sel. Evol.* 42:2. 10.1186/1297-9686-42-2 20105297 PMC2834608

[B4] Chu T. T. Alemu S. W. Norberg E. Sørensen A. C. Henshall J. Hawken R. (2018). Benefits of testing in both bio-secure and production environments in genomic selection breeding programs for commercial broiler chicken. *Genet. Sel. Evol.* 50:52. 10.1186/s12711-018-0430-x 30390619 PMC6215651

[B5] Chu T. T. Bastiaansen J. W. M. Berg P. Romé H. Marois D. Henshall J. (2019). Use of genomic information to exploit genotype-by-environment interactions for body weight of broiler chicken in bio-secure and production environments. *Genet. Sel. Evol.* 51:50. 10.1186/s12711-019-0493-3 PMC6751605 31533614

[B6] FAO (2000). *Management of Small Populations at Risk.* Available online at: http://www.fao.org/3/a-w9361e.pdf (assessed March 9, 2020)

[B7] Gowane G. R. Lee S. H. Clark S. Moghaddar N. Al-Mamun H. A. van der Werf J. H. J. (2019). Effect of selection and selective genotyping for creation of reference on bias and accuracy of genomic prediction. *J. Anim. Breed. Genet.* 136 390–407. 10.1111/jbg.12420 31215699

[B8] Hinrichs D. Meuwissen T. H. E. Ødegard J. Holt M. Vangen O. Woolliams J. A. (2007). Analysis of inbreeding depression in the first litter size of mice in a long-term selection experiment with respect to the age of the inbreeding. *Heredity* 99 81–88. 10.1038/sj.hdy.6800968 17519972

[B9] Howard J. T. Rathje T. A. Bruns C. E. Wilson-Wells D. F. Kachman S. D. Spangler M. L. (2018). The impact of selective genotyping on the response to selection using single-step genomic best linear unbiased prediction. *J. Anim. Sci.* 96 4532–4542. 10.1093/jas/sky330 30107560 PMC6247857

[B10] Jiménez-Montero J. A. González-Recio O. Alenda R. (2012). Genotyping strategies for genomic selection in small dairy cattle populations. *Animal* 6 1216–1224. 10.1017/S1751731112000341 23217224

[B11] Kause A. Ritola O. Paananen T. Wahlroos H. Mantysaari E. A. (2005). Genetic trends in growth, sexual maturity and skeletal deformations, and rate of inbreeding in a breeding programme for rainbow trout (Oncorhynchus mykiss). *Aquaculture* 247 177–187. 10.1016/j.aquaculture.2005.02.023

[B12] Legarra A. Reverter A. (2017). Can we frame and understand cross-validation results in animal breeding? *Proc. Assoc. Advmt. Anim. Breed. Genet.* 22 73–80.

[B13] Madsen P. Jensen J. (2013). *DMU: A User’s Guide. A Package for Analysing Multivariate Mixed Models, Version 6, Release 5.2.* Available online at: http://dmu.agrsci.dk/ (accessed September 12, 2018)

[B14] Mulder H. Bijma P. (2005). Effects of genotype × environment interaction on genetic gain in breeding programs. *J. Anim. Sci.* 83 49–61. 10.2527/2005.83149x 15583042

[B15] Patry C. Ducrocq V. (2011). Evidence of biases in genetic evaluations due to genomic preselection in dairy cattle. *J. Dairy Sci.* 94 1011–1020. 10.3168/jds.2010-3804 21257070

[B16] Pedersen L. Sørensen A. Henryon M. Ansari-Mahyari S. Berg P. (2009). ADAM: A computer program to simulate selective breeding schemes for animals. *Livest. Sci.* 121 343–344. 10.1016/j.livsci.2008.06.028

[B17] Su G. Sørensen A. C. Chu T. T. Meier K. Nielsen T. Lund M. S. (2020). Impact of phenotypic information and composition of reference population on genomic prediction in fish under the presence of genotype by environment interaction. *Aquaculture* 526:735358 10.1016/j.aquaculture.2020.735358

[B18] VanRaden P. M. Van Tassel C. P. Wiggans G. R. Sonstegard T. S. Schnabel R. D. Taylor J. F. (2009). Invited review: reliability of genomic predictions for North American Holstein bulls. *J. Dairy Sci.* 92 16–24. 10.3168/jds.2008-1514 19109259

[B19] Vitezica Z. G. Aguilar I. Misztal I. Legarra A. (2011). Bias in genomic predictions for populations under selection. *Genet. Res.* 93 357–366. 10.1017/S001667231100022X 21767459

[B20] Wang L. Janss L. L. Madsen P. Henshall J. Huang C.-H. Marois D. (2020). Effect of genomic selection and genotyping strategy on estimation of variance components in animal models using different relationship matrices. *Genet. Sel. Evol.* 52 1–14. 10.1186/s12711-020-00550-w 32527317 PMC7291515

