# Supplementary material for: Phenotypically Selective Genotyping Realizes More Genetic Gains in a Rainbow Trout Breeding Program in the Presence of Genotype-by-Environment Interactions
Source: Front Genet. 2020 Sep 11;11:866. doi: 10.3389/fgene.2020.00866 (PMC7517704; doi:10.3389/fgene.2020.00866)
Supplement: Supplementary file 1 [file Data_Sheet.pdf]

Appendix

Appendix 1: Sex ratio genotyping

At a constant number of fish genotyped in the breeding environment (B), the use of a certain sex ratio can optimize the intensity of selection and thus genetic gain. The sex ratio was derived deterministically for the schemes that used random genotyping and selective genotyping.

Selective genotyping

In schemes that used selective genotyping of top fish in B, parents were selected in two steps: phenotypic selection and then genomic selection. In other words, all parents and candidates for genomic selection had already undergone phenotypic selection. It was assumed that the intensity of phenotypic selection for males to be genotyped was the same as for females.

The accuracy of genomic breeding values (GEBV) was unaffected regardless of the sex ratio genotyped because the total number of genotyped fish for both sexes was constant. However, the genotyping of different sex ratios can affect the rate of genetic gain: that is, ΔG is proportional to $(i_s + i_d)$, where i_s is the intensity of genomic selection of sires from genotyped male candidates and i_d is the intensity of genomic selection of selected females from genotyped females. The intensity of selection of 50 sires for mating i_s was $i_s = f\left(\frac{50}{n_M}\right)$, where f() is the function for calculating the intensity of selection from the proportion of selected fish and n_M is the number of males in B genotyped per year and is an integer value greater than 50.

Note that i_d is the intensity of selection of the 400 females because the selection of 50 dams for mating from among the 400 selected females was random. The 400 females were selected from among 3-year-old female offspring in the current year and from among 4-year-old females that had been used as potential dams in the previous year. Two steps were involved in the selection of 4-year-old females. However, in its simplest terms, the intensity of selection of the 400 selected females i_d was approximated as $i_d \approx \frac{D_3}{400} f\left(\frac{D_3}{n_F}\right) + \frac{(D_4)}{400} f\left(\frac{D_4}{D_3}\right)$, where n_F is the number of females in B genotyped per year and is an integer value greater than D_3 or equal to 0, D_3 is the number of 3-year-old females selected from among the genotyped females, and $D_4 = 400 - D_3$ is the number of 4-year-old selected females selected from among the 3-year-old selected females in the previous year. The sum of n_M and n_F was 1000, 800, 600, and 400 when the breeding scheme allocated 0%, 20%, 40%, and 60%, respectively, of genotyping to fish in the commercial environment (C). When the proportion of selected females kept in two consecutive years was 11% and 100%, D_3 was 360 and 200, respectively, and D_4 was 40 and 0, respectively. When D_4 was 0, $i_d = \frac{D_3}{400} f\left(\frac{D_3}{F}\right)$.

To optimize the genetic gains of schemes that used selective genotyping, the sex ratio of genotyped individuals had to lead to the maximum of $(i_s + i_d)$:

$$max(i_s + i_d) = f\left(\frac{50}{n_M}\right) + \frac{D_3}{400} f\left(\frac{D_3}{n_F}\right) + \frac{(D_4)}{400} f\left(\frac{D_4}{D_3}\right). \quad \text{(A1)}$$

Because D_3, D_4, and the sum of n_M and n_F were known, we could identify n_M and n_F by varying the values. However, in practice, n_M and n_F cannot be implemented precisely. Thus, values of n_M and n_F that were multiples of 50 were used to find the values with ΔG optimized.

These procedures were used to determine n_M and n_F for the scenarios that used selective genotyping of top individuals. For the scenarios that used selective genotyping of top and bottom individuals, the procedures were similar except that the sum of n_M and n_F was equal to the number of top individuals genotyped. In a preliminary analysis, when the sum of n_M and n_F was assumed to be equal to the number of top and bottom individuals, genetic gains were lower, and the selection of bottom fish as parents was very rare.

Random genotyping

In schemes that used random genotyping of fish in B, the selection of parents could be based on either genomic selection for both males and females or the use of genomic selection for males and phenotypic selection for females. Genotyped individuals that were randomly selected from offspring were candidates for genomic selection. If no females were genotyped, potential dams were selected based on phenotypic selection. Therefore, genetic gains of the schemes could be from genomic selection or phenotypic selection. The genotyping of different sex ratios can affect the rate of genetic gain; that is, ΔG is proportional to $\left\{\rho_{gc}\frac{(i_{s1}+i_{d1})}{2}+\sqrt{h^2}r_g\frac{(i_{s2}+i_{d2})}{2}\right\}$, where ρ_{gc} is the accuracy of GEBV of the trait measured in C; h^2 is the heritability of the trait; i_{s1} and i_{d1} are the intensity of selection of sires and selected females based on GEBV, respectively; and i_{s2} and i_{d2} are the intensity of selection of sires and selected females based on phenotypic selection, respectively. We obtained the accuracy of GEBV by simulating the preliminary schemes that used random genotyping. The sex ratio of genotyped individuals of these preliminary schemes was same as the ratio used in the corresponding schemes that used selective genotyping.

It was assumed that

- When $n_M \le 50$, $i_{s1} = 0$ and $i_{s2} = f\left(\frac{1}{20}\right)$
- When $n_M > 50$, $i_{s2} = 0$ and $i_{s1} = f\left(\frac{50}{n_M}\right)$
- When $n_F \le D_3$, $i_{d1} = 0$ and $i_{d2} = f\left(\frac{1}{20}\right)$
- When $n_F > D_3$, $i_{d2} = 0$ and $i_{d1} = \frac{D_3}{400} f\left(\frac{D_3}{n_F}\right) + \frac{(D_4)}{400} f\left(\frac{D_4}{D_3}\right)$,

where n_M and n_F are the number of males and females in B genotyped per year, respectively; D_3 is the number of 3-year-old selected females selected from among the genotyped females; and $D_4 = 400 - D_3$ is the number of 4-year-old selected females selected from among the 3-year-old selected females in the previous year.

We varied n_M and n_F to find the values with ΔG optimized. Sires were genotyped in all scenarios, but females in B were not genotyped in some scenarios.

Appendix 2: Sex ratios genotyped for scenarios that used different genotyping of fish in B, proportions of genotyping allocated to fish in C (P_C%), proportions of selected females kept in two consecutive years (P_F%), genetic correlations (r_g) between the trait measured in B and C, heritability h^2 of the trait (h^2)

Genotyping strategy	P_C%	P_F%	h^2	r_g	Sex ratio genotyped (males:females)
Random	20	100	0.1	0.2	350:450
Random	20	100	0.1	0.5	350:450
Random	20	100	0.1	0.8	800:0
Random	20	100	0.3	0.2	350:450
Random	20	100	0.3	0.5	800:0
Random	20	100	0.3	0.8	800:0
Top1_1	0	11	0.3	0.2; 0.5; 0.8	400:600
Top1_1	0	100	0.3	0.2; 0.5; 0.8	400:600
Top1_1	20	11	0.3	0.2; 0.5; 0.8	300:500
Top1_1	20	100	0.3	0.2; 0.5; 0.8	350:450
Top1_1	40	11	0.3	0.2; 0.5; 0.8	600:0
Top1_1	40	100	0.3	0.2; 0.5; 0.8	250:350
Top1_1	60	11	0.3	0.2; 0.5; 0.8	400:0
Top1_1	60	100	0.3	0.2; 0.5; 0.8	400:0
T3_4B1_4	20	100	0.1; 0.3	0.2; 0.5; 0.8	250:350
T1_2B1_2	20	100	0.1; 0.3	0.2; 0.5; 0.8	800:0

Note: Genotyping of fish in B: random genotyping (Random),; selection of the phenotypically best fish from each sample of 20 fish (Top1_1),; selection of fish from three of the four top fish and one of the four bottom fish, while where the top and bottom fish were the best and worst fish, respectively, from each sample of 20 fish (T3_4B1_4);, selection of fish from one of the two top fish and one of the two bottom fish, where while the top and bottom fish were the best and worst fish, respectively, from each sample of 20 fish (T1_2B1_2). B, breeding environment; C, commercial environment.

Appendix 2 shows the sex ratios genotyped for all scenarios in our study. In some scenarios, no female offspring were genotyped; rather, females were selected based on phenotype in B. This was based on the assumptions that trout had have reproductive capacity and that phenotypic selection is a relatively inexpensive approach to improving the breeding program for the fish. In our study, family size was limited to 200, giving 20,000 offspring per year. This constant number of offspring was used for genotype testing of fish in B and C in all scenarios. In the scenarios in which no females were genotyped and 100% of selected females were kept in two consecutive years, each family produced an extra 40 offspring, giving 4000 female offspring for the phenotypic selection of 200 females as potential parents. In the scenarios in which no females were genotyped and 11% of selected females were kept in two consecutive years, each family produced an extra 70 offspring, giving 7000 female offspring for the phenotypic selection of 350 females as potential parents.

Appendix 3: Expected accuracy and bias of GEBV

In the scenarios with records measured in C, the accuracy of GEBV was calculated as the correlation between GEBV of the C trait and TBV of the C trait:

$$cor(GEBV_C, TBV_C) = \frac{cov(GEBV_C, TBV_C)}{\sqrt{var(GEBV_C)var(TBV_C)}} \quad \text{(A3.1)}$$

where $cor(GEBV_C, TBV_C)$ is the accuracy of GEBV in the scenarios with records measured in C; $cov(GEBV_C, TBV_C)$ is the covariance between GEBV of the C trait and TBV of the C trait; and $var(GEBV_C)$ and $var(TBV_C)$ are the variances of GEBV of the C trait and TBV of the C trait, respectively. In these scenarios, bias of GEBV (b_{GEBV_C,TBV_C}) was calculated as the regression slope of TBV of the C trait on GEBV of the C trait and was equal to:

$$b_{GEBV_C,TBV_C} = \frac{cov(GEBV_C, TBV_C)}{var(GEBV_C)} \quad \text{(A3.2)}$$

In our derivations of the expected accuracy of GEBV and expected bias of GEBV, we assumed that the GEBV of a trait is expected to be the same as the TBV of that trait. Therefore, in the scenarios with records measured in C, the expected accuracy of GEBV in (A3.1) was 1, and the expected bias of GEBV in (A3.2) was 1.

In the scenarios without records measured in C, the accuracy of GEBV was calculated as the correlation between GEBV of the B trait and TBV of the C trait:

$$cor(GEBV_B, TBV_C) = \frac{cov(GEBV_B, TBV_C)}{\sqrt{var(GEBV_B)var(TBV_C)}} \quad \text{(A3.3)}$$

where $cor(GEBV_B, TBV_C)$ is the accuracy of GEBV in the scenarios without records measured in C; $cov(GEBV_B, TBV_C)$ is the covariance between GEBV of the B trait and TBV of the C trait; and $var(GEBV_B)$ is the variance of GEBV of the C trait. In these scenarios, bias of GEBV (b_{GEBV_C,TBV_C}) was calculated as the regression slope of TBV of the C trait on GEBV of the C trait and was equal to:

$$b_{GEBV_B,TBV_C} = \frac{cov(GEBV_B, TBV_C)}{var(GEBV_B)} \quad \text{(A3.4)}$$

Equation (A3.4) can be reformulated:

$$b_{GEBV_B,TBV_C} = \frac{cov(GEBV_B, TBV_C)}{\sqrt{var(GEBV_B)var(TBV_C)}} \times \sqrt{\frac{var(TBV_C)}{var(GEBV_B)}} \quad \text{(A3.5)}$$

In the scenarios without records measured in C, the expected accuracy of GEBV in (A3.3) was equal to $cor(TBV_B, TBV_C)$ that is r_g. The expected bias of GEBV in (A3.4) and (A3.5) was $r_g \times \sqrt{\frac{var(TBV_C)}{var(TBV_B)}}$. As $var(TBV_C)$ and $var(TBV_B)$ were identical, and equal to 1, the expected bias of GEBV was r_g in the scenarios without records measured in C.

Appendix ~~3~~4: Variance components estimated from pedigree-based bivariate model for different genotyping scenarios for fish in B and C

Parameter	Simulated value	B-Random C-Random	B-Top1_1 C-Random	B-T1_2B1_2 C-Random	B-Top1_1 C-T1_1B1_1	B-Top1_1 C-Top1_1
$\sigma^2_{g_B}$	1	0.955	0.223	9.755	0.216	0.227
$\sigma^2_{g_C}$	1	0.957	0.91	0.908	8.488	0.221
r_g	0.5	0.453	0.198	1.323	0.446	0.121
$\sigma^2_{g_B}$	2.333	2.36	1.04	3.557	1.04	1.039
$\sigma^2_{g_C}$	2.333	2.381	2.416	2.369	2.865	1.042
h^2_B	0.3	0.288	0.176	0.733	0.172	0.179
h^2_C	0.3	0.286	0.273	0.276	0.747	0.173

Note: Pedigrees were registered for genotyped fish only. Genotyping strategies: random genotyping (Random), selection of fish from one of the two top fish and one of the two bottom fish while the top and bottom fish were the best and worst fish, respectively, from each sample of 20 fish (T1_2B1_2), selection of the phenotypically best fish from each sample of 20 fish (Top1_1), and selection of the phenotypically best and worst fish from each sample of 20 fish (T1_1B1_1). The direct genetic variance, residual variance, and heritability are $\sigma^2_{g_B}$, $\sigma^2_{e_B}$, and h^2_B, respectively, for the trait measured in B and $\sigma^2_{g_C}$, $\sigma^2_{e_C}$, and h^2_C, respectively, for the trait measured in C. The scenarios were simulated with r_g of 0.5, heritability of 0.3, 20% of genotyping allocated to fish in C, and 100% of selected females kept in two consecutive years. B, breeding environment; C, commercial environment.
